# A graphical analysis of aspects contributing to the spreading of measurements of left ventricular function

**DOI:** 10.1007/s10554-023-02796-z

**Published:** 2023-02-17

**Authors:** Christian Knackstedt, Georg Schummers, Jörg Schröder, Nikolaus Marx, Joost Lumens, Sandra Sanders-van Wijk, Bram Ramaekers, Michael Becker, Vanessa van Empel, Hans-Peter Brunner-La Rocca, the PsychEcho study group

**Affiliations:** 1 Department of Cardiology and Cardiovascular Research Institute Maastricht (CARIM), Maastricht University Medical Center+, 6202 AZ Maastricht, The Netherlands; 2 TOMTEC Imaging Systems GmbH, Unterschleissheim, Germany; 3 Department of Cardiology, Angiology, Pneumology and Intensive Care Medicine, RWTH Aachen University Hospital, Aachen, Germany; 4 Department of Biomedical Engineering, Cardiovascular Research Institute Maastricht (CARIM), Maastricht University, Maastricht, The Netherlands; 5 Department of Cardiology, Zuyderland Hospital, Heerlen, The Netherlands; 6 Department of Clinical Epidemiology and Medical Technology Assessment, Maastricht University Medical Centre+, Maastricht, The Netherlands; 7 Department of Cardiology, Rhein-Maas Klinikum, Würselen, Germany

**Keywords:** Ejection fraction, Eechocardiography, Left ventricular function, Observer variability

## Abstract

The Simpson’s method is the standard technique to determine left ventricular (LV) ejection fraction (EF) on echocardiography. The large inter-observer variability of measuring LVEF is well documented but not fully understood. A graphical analysis was used to elaborate what contributes to the inter-observer difference. Forty-two cardiologists (32 male, 39 ± 7 years) evaluated the LVEF using the Simpson’s method on 15 different echocardiograms (2 and 4 chamber view (2CH/4CH)); the program did not show the result of EF to prevent a bias. End-diastolic (ED) and end-systolic (ES) frames were predefined ensuring measurement at the same time point of the cardiac cycles. After standardization of the LV contour, the differences of the individual contours compared to a reference contour were measured. Also, the spreading of lateral/medial mitral annulus contours and the apex were depicted. A significant spreading of LV-contours was seen with larger contours leading to higher EFs (p < 0.001). Experience did not influence the determination of LVEF. ED-volumes showed more spreading than ES-volumes ((3.6 mm (IQR: 2.6–4.0) vs. 3.4 mm (IQR: 2.8–3.8), p < 0.001). Also, the differences were larger for the 2CH compared to the 4CH (p < 0.001). Variability was significantly larger for lateral than septal wall (p < 0.001) as well as the anterior compared to the inferior wall (p < 0.001). There was a relevant scattering of the apex and medial/ lateral mitral annulus ring. There was a large variability of LV-volumes and LVEF as well as position of mitral valve ring and apex. There were global differences (apical 2CH or 4CH), regional aspects (LV walls) and temporal factors (ED vs. ES). Thus, multiple factors contributed to the large variability.

**Trial registration**: The study was registered at “Netherlands Trial Register” (www.trialregister.nl; study number: NL5131).

## Introduction

The determination of left ventricular (LV) ejection fraction (EF) is one of the most important parameters in cardiology. Many therapeutic strategies e.g. in patients with heart failure (HF) are based on a certain LVEF threshold [1].

LVEF can be analyzed using different imaging techniques but echocardiography remains the most applied one [2, 3]. However, many studies have shown a clinically relevant inter- and intra-observer variability of echocardiographic LVEF measurement [4–8]. This is in line with other echocardiographic measurements such as septal thickness [9]. This carries clinical consequence as re-assessment of LVEF or septal thickness might lead to a re-allocation of patients with respect to certain therapies [5, 9]. Still, the determination of LVEF and its inter-observer variability has never been investigated in a large group of cardiologist [5, 7, 8]. There might be certain aspects (e.g. positioning of the apex) or depiction of regional segments that influence variability the most. A quantitative analysis of the observer-related sources of LVEF variability may help to improve training and standardization of echocardiographic assessment of LVEF.

The purpose of this study was to evaluate sources of LVEF measurement variability using a graphical analysis.

## Methods

### Study cohort

Convenience sampling was used to identify cardiologists known to two of the organizers (CK, HPB). 46 physicians responded and 42 (Netherland: 31 and Germany: 11 physicians) were included (32 (76%) male, mean age: 39 ± 7 years). Results from four cardiologists were excluded due to technical problems during data acquisition that could not be solved. Importantly, all participants had passed their clinical rotation in the echocardiography lab to ensure adequate experience. In addition, all participants, except cardiologists, had to be found capable using a standardized “entrustable professional activities” approach and reaching level 3 [10].

The following characteristics of the participating cardiologists were collected using a questionnaire: age, gender, position at institution, years practicing as cardiologist, cardiology subspecialty, years performing echocardiograms, experience in other imaging modalities (e.g. computed tomography, cardiac magnetic resonance imaging).

The majority of participants worked as residents (in training to become cardiologist) (18; 43%), others as attending cardiologists (16; 38%), one as fellow (1; 2%) or some as director of departments/ sub-departments (4; 10%); three individuals did not provide their status (3; 7%). The median work experience as cardiologist (training period not included, n = 21) was 5 years (IQR: 0–8). The median time span performing echocardiograms was 6.5 years (IQR: 3–12). There was a broad distribution of different sub-specialties: interventionalists (6; 14%), electrophysiologists (6; 14%), intensive care specialists (3; 7%), imaging specialists (8; 19%), heart failure specialists (8; 19%). Almost half of the physicians (20; 48%) had experience in other imaging techniques.

### Study organization

A list containing the name and contact information of the physicians was given to GS from TOMTEC Imaging Systems having several functions: (1) providing a specific study code to each individual cardiologist which was used for labeling study material sent to the physicians, (2) technical support if needed, (3) receiving the return envelops and gathering data in a database and (4) sending back the pseudonymized results to the organizers of the study (CK, HPB). Importantly, the list containing names and individual study keys was not accessible to anybody except GS (neither the participating cardiologist nor the investigators). This approach was approved by the local ethics committee of Maastricht UMC + (study ID METC 15-4-151) to guarantee anonymity and discretion. The study was registered at “Netherlands Trial Register” (www.trialregister.nl*;* study number: NL5131).

Accordingly, all physicians received an envelope containing the following: (a) informed consent form, (b) short inventory regarding the personal status, (c) USB-drive with the program LV2D for LVEF evaluation, (d) document containing a detailed stepwise instruction how to perform the analysis and (e) return envelope (to be sent back to TOMTEC Imaging Systems GmbH).

### Evaluation of EF

The LV2D prototype (LV2D, TOMTEC Imaging Systems) was used for contouring of the LV needed for the Simpson analysis. The program included a database consisting echocardiograms of 15 patients with a two-chamber view (2CH) and a four-chamber view (4CH). Those were selected from the echocardiogram database of the MUMC + Maastricht by CK and HPB. They were obtained by different sonographers and were not done with contrast. Details can be found in Table 1. They were anonymized and then stored onto an USB-drive. The whole range of LVEF (from severely reduced to normal) was present. Also, the intention was to cover a realistic range of image quality. Therefore, the selection was controlled by CK and HPB and scored as follows: 1 = excellent, 2 = good, 3 = fair, 4 = bad/ not analyzable. For the 2CH, the image quality was as follows: 1: 4x, 1–2: 4x, 2: 5x, 2–3: 2x. Accordingly, for the 4CH the ratings were: 1: 4x, 1–2: 2x, 2: 8x, 3: 1x.

**Table 1:** Relevant clinical patient data

Age [years]	59 (IQR: 54–62)
Males	12 (80%)
Height [cm]	170 (IQR: 167–176)
Weight [kg]	70 (IQR: 65–83)
Body mass index [kg/m^3^]	23.7 (IQR: 22.5–28.70)
Body surface area [m^2^]	1.9 (IQR: 1.74–1.98)
Medical history	
Any cardiac disease	15 (100%)
Arterial hypertension	4 (73%)
Coronary artery disease	14 (93%)
Myocardial infarction	12 (80%)
COPD	2 (13%)
Diabetes mellitus	2 (13%)

Cardiologists were asked to start the LV2D program, select a patient and start the measurement tool. Due to study reasons, the analysis was performed on personal computers. This was the only way to perform the study but this manner could have potentially influenced the results.

A pre-defined end-diastolic (ED) and end-systolic (ES) frame was displayed for analysis. Also, a loop of the whole heart cycle was shown as a moving image. All segments that were not traced in the correct frame were excluded from later segmental analysis. Of note, the program did not display the actual result of the Simpson measurement; only the drawn contours were visible. The idea was to prevent adjustment of the contours based on the visual assessment.

### Segmental analysis of LV contours

The following steps were applied for analysis:

1. For all individual contours (2CH/4CH and ES/ED), equidistant resampling was performed using 99 points to achieve comparability between echocardiograms as the length of the LV differs in individual patients.
2. All contours of all cardiologists of *a single echocardiogram* were superimposed. This was done separately for 4CH (Fig. 1a) and 2CH as well as ES/ED. Then, *all 15 echocardiograms* were registered obtaining four images: 2CH-ED and 2CH-ES as well as 4CH-ED and 4CH-ES.

**Fig. 1:**
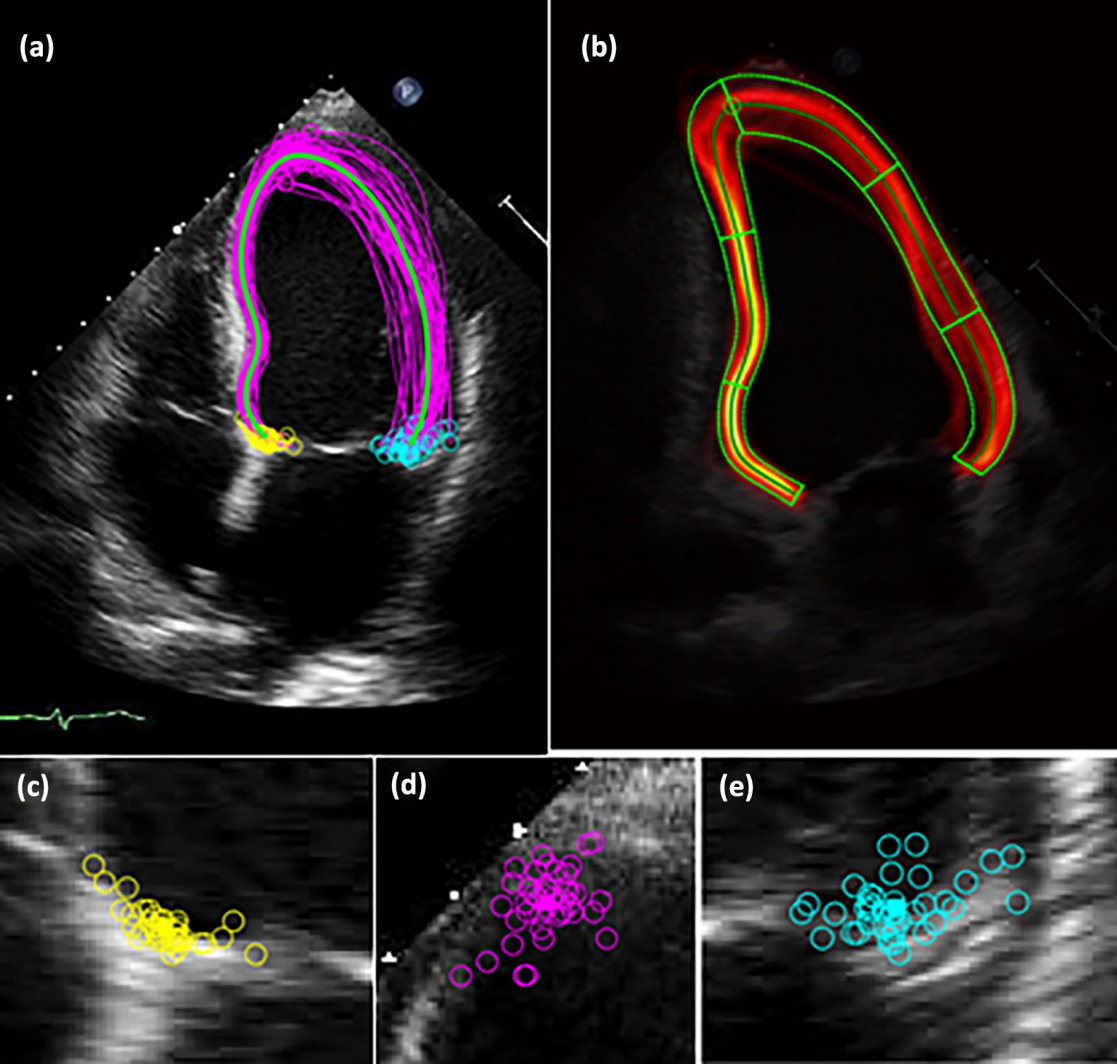
**a**–**e** Graphical analysis of measurement deviation on a 4-chamber view. All cardiologists were asked to draw contours for 15 four chamber view on an end-diastolic and end-systolic image. Then, all individual contours were combined on one echocardiogram. Also, the reference contour of the group was calculated. The images display the following: **a** 4-chamber view individual end-systolic contours (pink are the individual contours, blue is the reference contour), **b** 4-chamber view with depiction of distance between individual contours and reference contour per segment regarding end-systolic contours, **c** positioning of the posterior mitral valve ring, **d** positioning of the apex, **e** positioning of the anterior mitral valve ring
3. As comparison, a reference contour (according to the density/ “heat map” of the individual contours) for all 99 measuring points of the 15 echocardiograms/ all participants was calculated (both, 2CH/4CH and ED/ES).
4. The distance between each individual measurement and the reference contour on each standardized measuring point was calculated.
5. For analysis, 2CH and 4CH were divided into 6 segments defining the adjusted length (step 1) of the LV into six comparable segments (Fig. 1b). Consequently, 12 segments for comparison were obtained. In addition, this step was performed for ED and ES.
6. For comparison of different wall regions, segments were combined for the septal/lateral walls (4CH) as well as the inferior/anterior walls (2CH).
7. The median distance between each measurement and the reference contour was calculated *per segment* as well as *per wall region*. Finally, the values obtained from the steps above were combined to a median value for all echocardiograms for a standard 2CH and 4CH (Fig. 2).

**Fig. 2:**
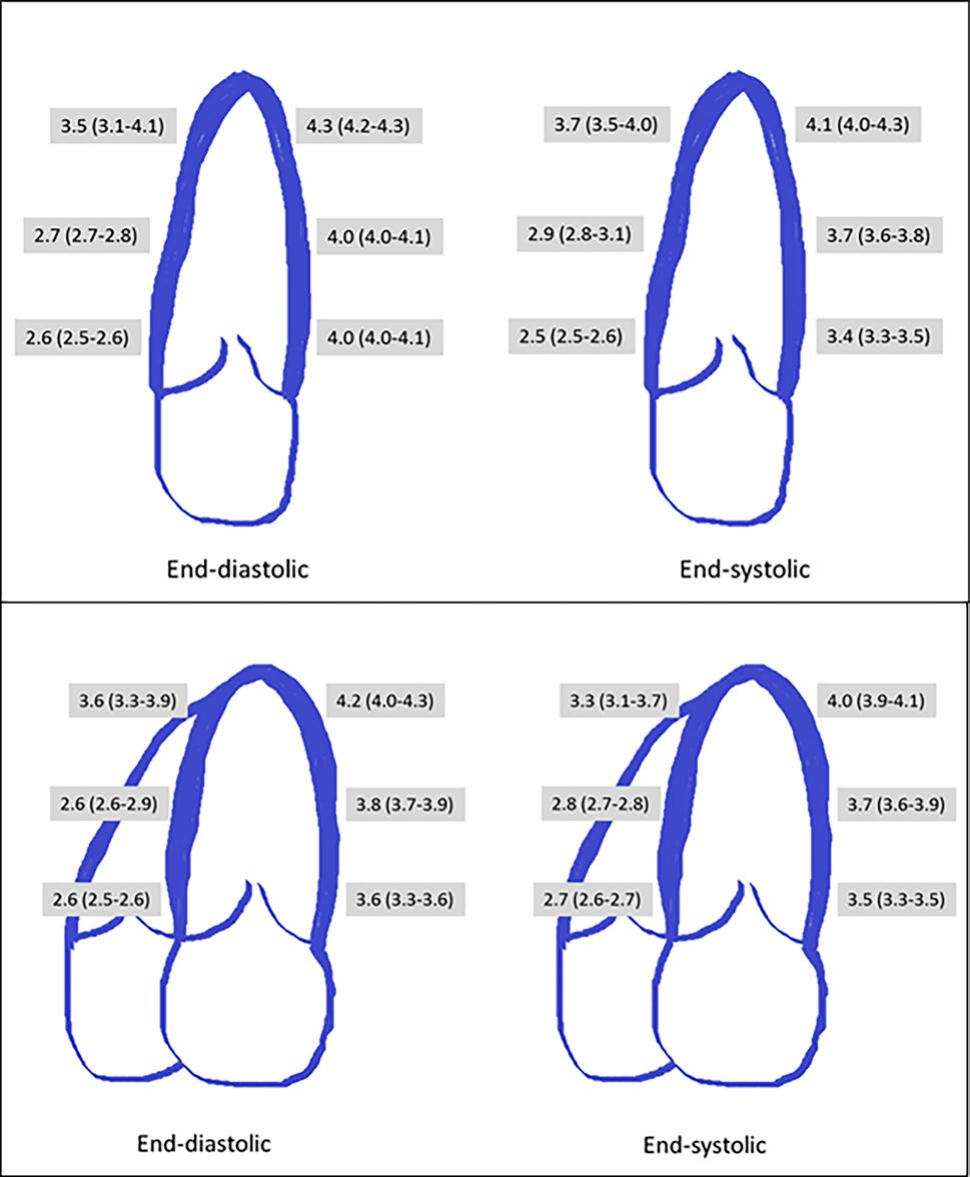
Depiction of the median distance between reference and individual contour per segment. For left ventricular segments, the distance [mm] (median ± IQR) between individual contour and reference contour was depicted. This was done for 2-chamber view (upper panel) and 4-chamber view (lower panel), for end-diastole and end-systole

### Analysis of the position of the apex

1. For 2CH and 4CH separately, the apex is determined as the point with the largest Euclidean distance to the center of the two mitral valve annuli based on the users’ contour. Both contour segments between annuli and apex (i.e. a) distance between septal annulus and apex and b) distance between lateral annulus and apex) are equidistantly resampled. The resulting standardized contour ensures that the indices 1 and 99 refer for both annuli and index 50 for the apex, see step 1 above). This was done for all 15 echocardiograms separately (Figs. 1 and 3).

**Fig. 3:**
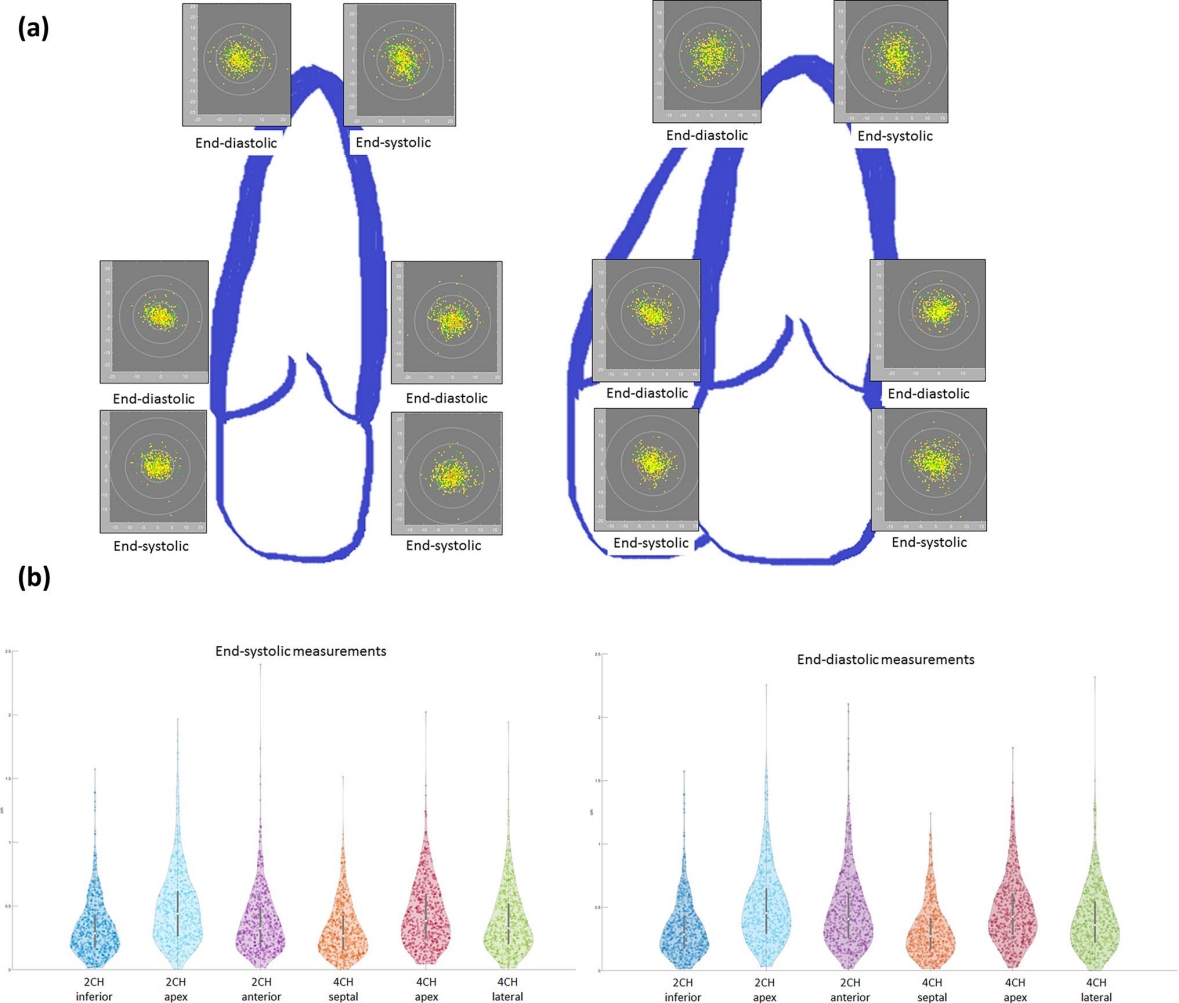
**a** Graphical depiction of the spreading of distinct anatomical landmarks. This figure displays the individual location of the apical and mitral valve contour (both, end-diastolic and end-systolic and 4-chamber view (left) and 2-chamber view (right)). For illustration, the reference position of all measurements is defined and the distance [mm] of the individual measurements calculated from that point (x-axis: horizontal image plane, y-axis: vertical image plane). The inner ring covers 1cm^2^, the following 4cm^2^ and the outer ring 9cm^2^. Image quality is also displayed to illustrate the influence on landmark position: green = excellent, yellow = good, orange = fair. **b** Violin plot of the spreading of distinct anatomical landmarks. This figure shows violin plots (depiction the distribution of measurements and markers for the median/ interquartile range) for the distance [cm] between reference position and individual location of the mitral valve ring and apical contour (both, end-systolic/ left and end-diastolic/ right)
2. A graphical depiction was performed combining all 15 echocardiograms (2CH and 4CH separately) (Fig. 3).
3. For the complete depiction, 2CH and 4CH were aligned in 3D. The center point of all apical contours was defined in 3D for all information obtained from 2 and 4CH.

### Analysis of the lateral/ septal or inferior/ anterior mitral ring position

1. For 2CH and 4CH separately, the contour of the lateral and the septal mitral valve annulus was noted (see step 1 above, measuring point 1 and 99 respectively on standardize contour). This was done for all 15 echocardiograms (Figs. 1 and 3).
2. More specifically, some colleagues did not draw a u-shaped contour for the LV but a whole circle. In those cases, the contours had to be adjusted to as both mitral annulus were connected. This was done in consensus by CK and GS.
3. Then, a graphical depiction was performed for septal and lateral annulus. Again, this was done for all 15 echocardiograms and both views (3).

### Statistical and graphical analysis

**Fig. 4:**
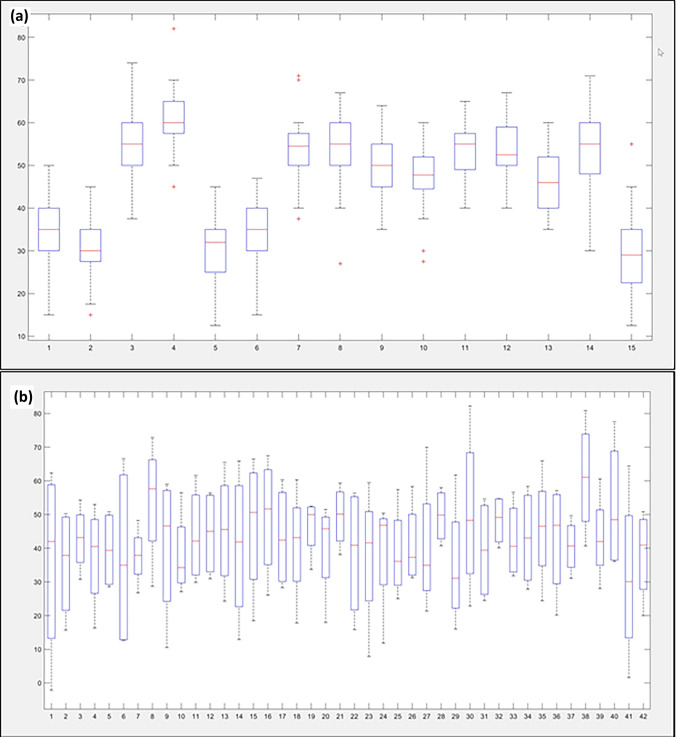
Depiction of the left ventricular ejection fraction based on Simpson measurement. The left ventricular ejection fraction measured using Simpson’s method on echocardiogram was displayed (box plots indicating: median with 25th/75th percentiles). This was done per **a** echocardiogram (1–15) and **b** per cardiologist (1–42)

**Fig. 5:**
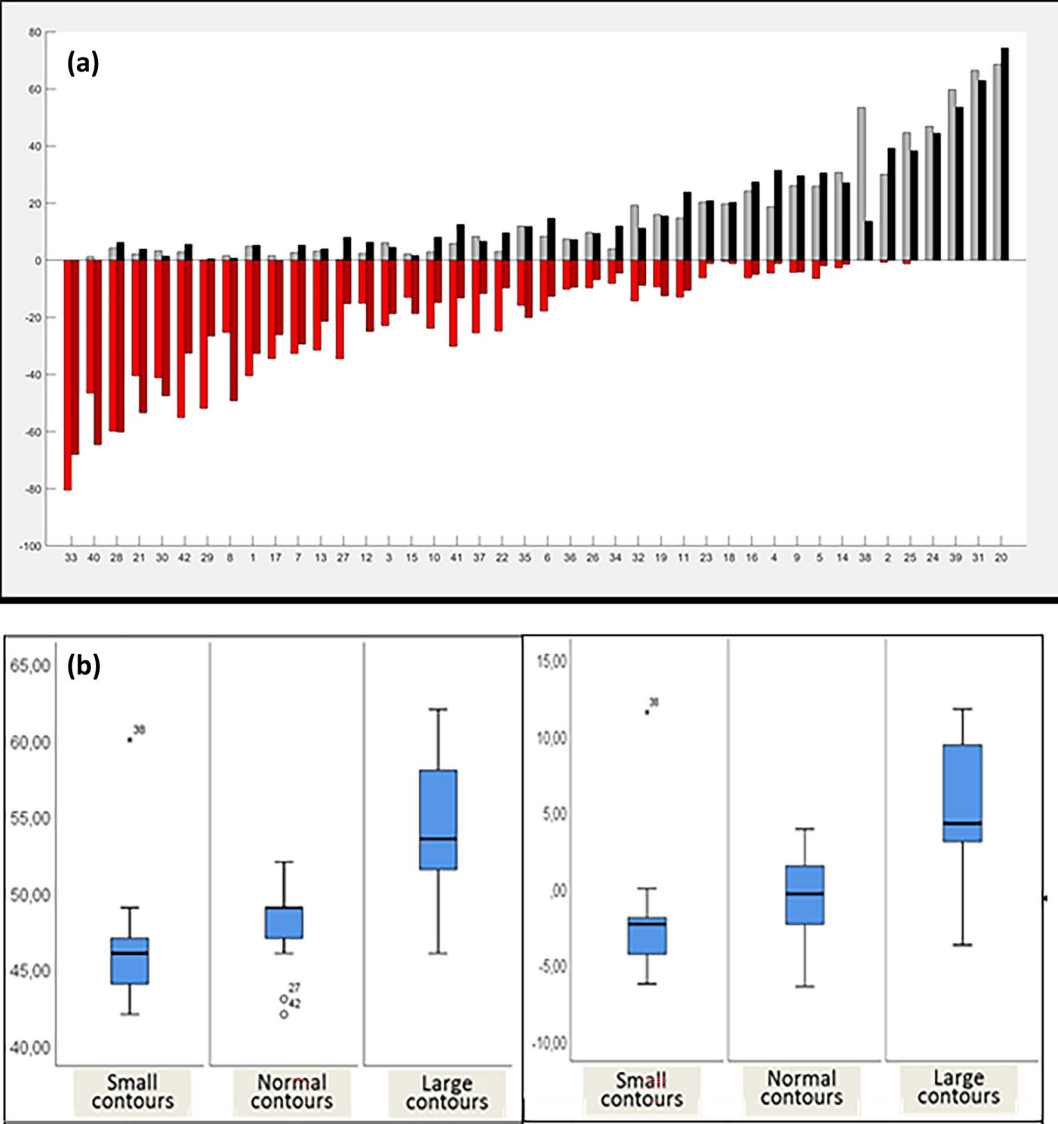
**a** Depiction of left ventricular end-diastolic and end-systolic by different cardiologists. This figure displays the difference between left ventricular volumes measured by individual cardiologists and the median of the group. The x-axis depicts the individual cardiologist, the y-axis the percentage of underestimation (negative deflection) or overestimation (positive deflection). Light red/ light grey shows the difference in end-diastolic volume (combining 2-chamber view and 4-chamber view), dark red/ dark grey depicts end-systolic volume (again, combining 2-chamber view and 4-chamber view). **b** Depiction of the left ventricular ejection fraction depending on tracing behavior. The relation between left ventricular (LV) ejection fraction and the location of the contours compared to the reference contour of the group is displayed. All individual cardiologists were allocated to three different groups based on the localization of their drawn contours: (1) small contours of the LV compared to the reference contour of the group (n = 13), (2) contours in line with the reference contour of the group (n = 21) or (3) contours larger than the reference contour of the group (n = 8). *Left Panel:* the median LV ejection fraction of the group was determined as well as (*right panel*) the median of the individual difference compared to the reference contour. The was a significant difference (LVEF: p < 0.001, difference to reference contour: p = 0.001) comparing the three groups regarding both measurements

All contouring including calculation of volumes and LVEF was done with MATLAB9.9. This holds also true for all visualization of measurements. More specifically, for LV volumes the discrepancy between volumes contoured by individual cardiologists was calculated (Fig. 4). Under- as well as overestimation was depicted for ED-LV (combining 2CH and 4CH) and ES-LV volumes (combining 2CH and 4CH) (Fig. 5a). In addition, the overall localization of individual LV contours was compared to the reference contour of the group. All individual cardiologists were allocated based on the localization of their contours: (1) small contours of the LV compared to the reference contour of the group (n = 23), (2) contours in line with the reference contour of the group (n = 11) or (3) contours larger than the reference contour of the group (n = 8). Then, the median LVEF of the group was determined as well as the median of the individual difference from the group (Fig. 5b). Of note, this classification took place without knowing the EF results.

Characterization of the population was done using descriptive statistics: quantitative data is expressed as mean ± 2SD or median ± interquartile ranges as appropriate. Physicians were categorized by years of echocardiography experience: < 5 years (n = 17), 5–10 years (n = 13) or > 10 years (n = 12).

Groups (based on experience, localization of contours) were compared regarding the median LVEF using an Independant- Samples Kraskul Wallis Test (Fig. 5b). Comparison between regions (inferior, septal, lateral and anterior wall) was performed using the Related Samples Friedman’s two way analysis with pairwise comparison (for two regions). P-values < 0.05 were considered statistically significant. Correlations were calculated using the Spearman coefficient. Statistical analysis was performed with a PASW software package (PASW Statistic version 25).

## Results

### Comparison of EF, ED and ES volumes per cardiologist

The ED-LV volume (biplane) was 125 ± 41 ml and ES-LV volume (biplane) was 68 ± 34 ml. The mean biplane LVEF was 49 ± 15% (Fig. 4a and b).

Lower ED-LV volumes were related with lower ES-LV volumes (Fig. 5a) (r = 0.8868). In addition, grouping physicians according to their contouring behavior, larger contours were related to higher EFs (p < 0.001). This held true for the absolute EF (small contours (n = 13): 46% (43.5–47), medium contours (n = 21): 49% (46.5–49.5) and large contours (n = 8): 53.5% (51.3–59)) and the difference compared to the reference contour (small contours (n = 13): − 2.4% (− 5–(− 1.8)), medium contours (n = 21): -0.4% (− 2.4–1.6) and large contours (n = 8): 4.2% (2.9–10.5)) (p < 0.001) (Fig. 5b).

There was a higher correlation coefficient between the ES-LV volume and the EF (r = − 0.8363) compared to the ED-LV volume (r = − 0.5113).

There was no difference between categories according to experience regarding absolute EF (p = 0.998) or relative EF differences (p = 0.990). The median EF was 49% (IQR: 46–51.5) for colleagues with < 5 year (n = 17), 48% (IQR: 46–51) with 5–10 years (n = 11) and 48% (IQR: 46–51) > 10 years of experience (n = 14). The same held true for the relative EF difference from reference EF: − 1% (IQR: − 3.7–3.2), − 1.1% (IQR: − 2.4–0.8) and − 1.2% (IQR: − 2.97–2.7).

### Comparison between individual vs. reference contour

Overall, there was a median difference between contours and the reference contour of 3.6 mm (IQR: 2.8–3.9).

Combining systolic and diastolic contours, there was a larger difference (individual vs. reference contour) for the 2CH (3.6 mm (IQR: 2.8–4.0) compared to the 4CH (3.4 mm (IQR: 2.7–3.8) (p < 0.001). Looking at systolic contours only, the range of 2CH contours was 3.5 mm (IQR: 2.9–3.8) and for the 4CH 3.4 mm (IQR: 2.7–3.8) (p < 0.001). Regarding the diastolic contours, 2CH showed a spreading of 3.9 mm (IQR: 2.7–4.1) whereas for the 4CH, this distance was 3.6 mm (IQR: 2.6–3.9) (p < 0.001).

When comparing different regions/LV walls, there was a significant difference regarding the mean measuring band width (p < 0.001). More specifically, difference was significantly larger for lateral as compared to septal wall (p < 0.001) as well as larger for anterior than inferior wall (p < 0.001). This held true for both ED and ES contours (p < 0.001). Figure 2 and Table 2 depicts those differences per segment.

**Table 2:** Depiction of the median difference for the individual contour compared to the reference contour

	2CH ED	2CH ES	4CH ED	4CH ES
All segments (median, IQR) [mm]	3.9 (2.7–4.1)	3.5 (2.9–3.8)	3.6 (2.6–3.9)	3.4 (2.7–3.8)
Anterior	4.1 (4.0 – 4.2)	3.7 (3.5 – 4.0)		
Inferior	2.7 (2.6 – 3.2)	2.9 (2.6 – 3.5)		
Septal			2.7 (2.6 – 3.3)	2.8 (2.7 – 3.1)
Lateral			3.8 (3.6 – 4.0)	3.7 (3.5 – 3.9)
Apical segments (median, IQR) [mm]	4.2 (3.5—4.3)	4.0 (3.7—4.2)	3.9 (3.6—4.2)	3.8 (3.3—4.0)
Mid segments (median, IQR) [mm]	3.5 (2.7—4.0)	3.5 (2.9—3.7)	3.3 (2.6—3.8)	3.2 (2.8—3.7)
Basal segments (median, IQR) [mm]	3.4 (2.6—4.1)	2.9 (2.5—3.4)	3.1 (2.6—3.6)	2.9 (2.7—3.5)
p (segments apical-basal)	< 0.001	< 0.001	< 0.001	< 0.001

This is done for different regions (apical, mid and basal segments as well as anterior, inferior, septal and lateral) as well as different point in time (end-diastolic as well as end-systolic) and apical two-/ four chamber view; 2CH = two chamber view, 4CH = four chamber view, ED = end-diastolic, ES = end- systolic

Overall, there was a larger median difference between individual contours and the reference contour for the ED of 3.6 mm (IQR: 2.6–4.0) compared to the ES of 3.4 mm (IQR: 2.8–3.8) (p < 0.001). Also, this difference was seen for the 2CH (ED: 3.9 mm (IQR: 2.7–4.1) vs. ES (3.5 mm (IQR: 2.9–3.8), p = 0.003) as well as the 4CH (ED: 3.6 mm (IQR: 2.6–3.8) vs. ES (3.4 mm (IQR: 2.8–3.8), p = 0.028).

Differentiating between apical, mid and basal segments, there was a significant difference between individual contours and the reference contour (p < 0.001). This was seen for ED but also ES contours for, both 2CH and 4CH (Table 2).

### Analysis of mitral valve and apical region

**Fig. 6:**
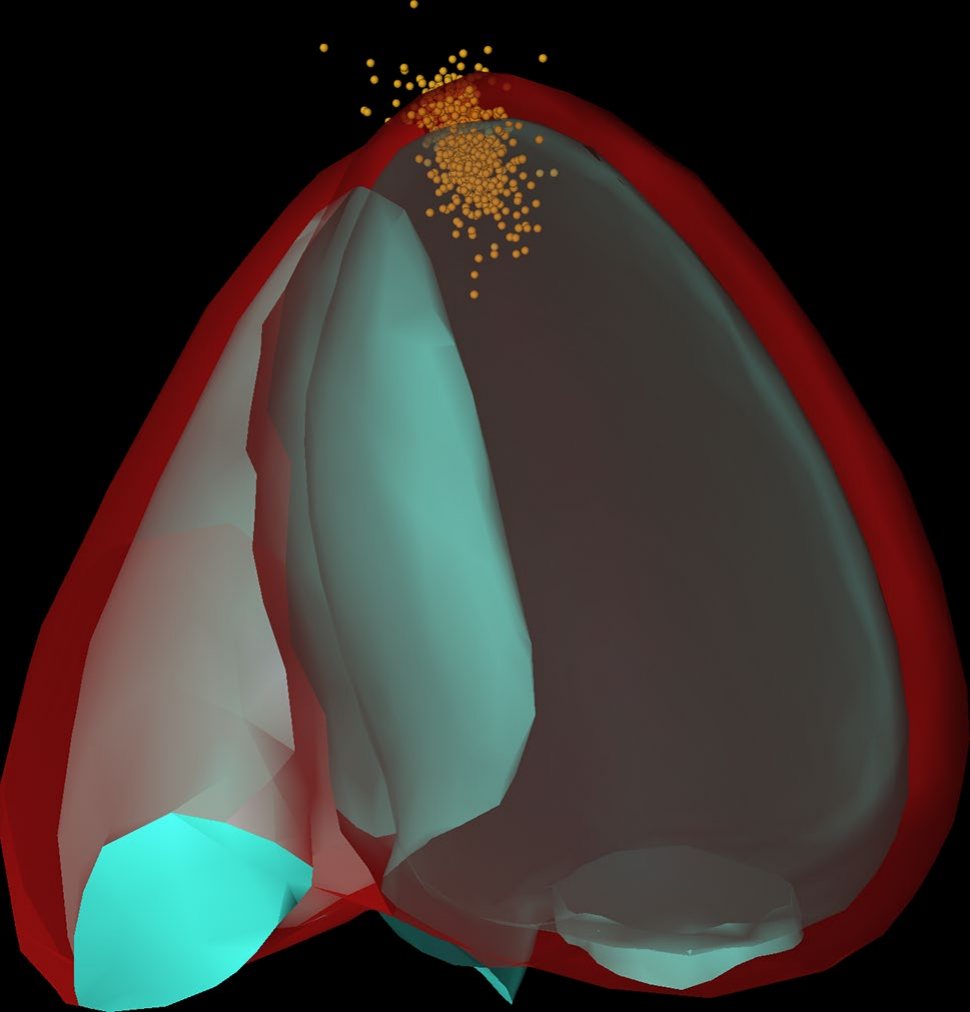
Depiction of all apical contours of the left ventricle in a three-dimensional model. This figure displays the localization of the apex based on individual positioning in three dimensions. More specifically, the apical localizations in the 2-chamber view and 4-chamber view were registered to determine a three-dimensional position. The length of the different left ventricle were standardized and superimposed on a heart model. Then, the apical position was noted (as yellow dots) on that cast

Also, the mitral and lateral mitral valve ring contours were placed in a relatively large region (Fig. 4). Within an area of 1 cm^2^, about 85% of the septal/ inferior mitral valve ring position was found. This was less for the lateral/ anterior ring and even less for the apical position (Table 3). This large spreading of the apical position is depicted in a three dimensional manner (registration of 2CH and 4CH contours) (Fig. 6).

**Table 3:** Depiction of the regional distribution of anatomical landmark position

Area	2CH ES			4CH ES		
	Inferior MVR	Apex	Anterior MVR	Septal MVR	Apex	Lateral MVR
< 1 cm^2^	85.6	66.1	81.5	87.4	71.5	80.3
< 4 cm^2^	13.4	30.1	17.5	12.4	27.2	18.4
< 9 cm^2^	1.0	3.0	0.7	0.2	1.2	1.2
> 9 cm^2^	0.0	0.8	0.3	0.0	0.2	0.2

Area	2CH ED			4CH ED		
	Inferior MVR	Apex	Anterior MVR	Septal MVR	Apex	Lateral MVR
< 1 cm^2^	85.6	64.7	70.5	86.4	69.1	77.2
< 4 cm^2^	13.4	31.3	25.9	13.5	27.9	20.6
< 9 cm^2^	1.0	3.8	3.0	0.2	2.8	2.0
> 9 cm^2^	0.0	0.2	0.7	0.0	0.2	0.2

Compared to the central reference position, the localization of the individual contour is depicted. More specifically. The percentage of individual markers within an area of < 1 cm^2^, < 4 cm^2^, < 9 cm^2^, > 9 cm^2^ is calculated. This is done for different landmarks (apical position, both mitral valve ring position) and for both apical views (two-/ four chamber view); *2CH * two chamber view, *4CH *four chamber view, *ED * end-diastolic, *ES * end- systolic, *MVR *mitral valve ring

## Discussion

This is the first study trying to identify possible contributing factors for the variability of Simpson’s biplane LVEF measurement based on (a) a graphical depiction of contours of (b) a large group of cardiologists. Several global, local and temporal aspects could be depicted implying that there is probably no single, simple solution to improve variability: a larger variability for 2CH compared to 4CH, diastolic contours larger than systolic contours, septal less variability than lateral/ inferior less than apical. In addition, apical and mitral valve ring position added to the spreading.

### Observer variability

Overall, our findings of large inter-observer variability of echocardiographic measurement of LVEF and LV volumes using different methods e.g. visual assessment or Simpson’s method are not new [5, 6, 8, 11]. Also, variability has been investigated in many clinical scenarios including cardiac diseases e.g. hypertension, myocardial infarction or aortic stenosis [12–14]. Still, our findings are not totally comparable as physicians were not able to readjust contours according to results of the Simpson’s method; this might have induced a somewhat artificial situation.

### Differences due to anatomical regions

Probably, every echocardiographer shares the impression that the cardiac septum is better visualized compared to the lateral wall [16]. This holds true for the inferior compared to the anterior wall. Indeed, we saw a significant difference between septal and lateral as well as inferior and apical wall regarding the band width of contouring. It could be speculated that the angle between the echo beam and left ventricular structures/ walls makes the difference. However, in a classical 2CH and 4CH, lateral wall and septum are equally positioned in relation to the echo beam [2].

### Clinical implication

2D echocardiography remains the standard approach in clinical practice as shown in a recent survey, whereas 3D and deformation imaging are only performed in the minority of patients [15]. Consequently, observer variability has relevant implications for clinical decision making as there was a e.g. relevant re-allocation of ICD indication due to two different measurements of LVEF [5]. Recently, it was nicely demonstrated that, even in a group of highly trained physicians with high quality images, there was a large variability of septal thickness leading to differences in risk stratification in patients with hypertrophic cardiomyopathy [9].

We do not intend to say that echocardiography is the wrong method to determine LVEF as it will remain the main measurement until more experience is gathered using a more individual, precision based approach [20, 21]. However, the dependence on the human factor should be placed into focus as these findings hold true for the interpretation of many other parameters on echocardiography e.g. diastolic function, septal thickness or stress-echocardiography [9, 17, 18]. Also, reliable recognition of subtle wall motion abnormalities i.e. hypokinesia might be difficult because of subjectivity even in expert readers [11]. This problem becomes even more complex as there are more issues e.g. scan-rescan variability and intra- observer variability. Those components might add up to more clinical relevance but were not investigated in the current study [22].

The central question is, what do we find acceptable as variability? The evidence regarding the inter-/ intra-observer variability of large core labs dealing with multicenter studies is sparse [19]. Even though highly experienced readers were evaluated, percentage of LVEF measurements within a range of 5% ranged from 43 to 54% between different imaging modalities [19]. There are two excellent studies that looked into the placement of contours/ measurement markers and the inter- reader differences on echocardiography [9, 22]. Using the best available images and experienced readers, there was a relevant discrepancy regarding the measurement of septal thickness in patients with hypertrophic cardiomyopathy as shown by Captur et al. [9]. They found an intra-modality intra-reader variability (duplicate data sets, all readers) of 2.1 mm [95% CI 1.7–2.6 mm] as well as an intra-modality inter-reader variability (same data sets, different readers) of 4.63 mm [95% CI 2.22 mm–7.05 mm]. Using a 3D model, Mor-Avi et al. demonstrated that on phantom studies that the exact boundary position was crucial for accurate volume measurements. A difference of 1 mm in surface position lead to a considerable differences in the calculated volumes [22]. As our evaluation was based on 2D images, we cannot directly compare the results. Still, a difference of 1 or even 2 mm implies a relative different. However, the main question remains whether this is clinically feasible. This is underlines by the fact that we saw larger variation at e.g. the lateral wall.

### Potential, future solutions

The main challenge is rather how we can improve the method and overcome shortcomings in clinical practice. Unfortunately, education does not seem to be the key as seen in this study but also found by other groups [9, 17, 28, 29]. Initially, there is a significant learning curve regarding the visual estimation of LVEF applying different methods. At the end, a relevant inter-observer variability remained [17, 28, 29]. Also, we could not show any influence of experience regarding the difference between individual contours and the reference contour of the group. There have been many attempts to improve observer variability by the use of echo contrast conflicting results [7, 27]. However, echo contrast does not belong to daily routine in all echo labs as pointed out recently [15]. Imaging quality due to the use of other or newer echocardiographic probes might have an impact, too. To our knowledge, there are no studies investigating that in a standardized manner.

Obviously, one of the drawbacks of the two-dimensional Simpson’s method is the need of geometric assumptions. Therefore, three-dimensional imaging seems to be the better approach [22–25]. This also means that there is less modification due to variation in imaging planes between different acquisitions. Also, 2D can problematic as there might be foreshortening of the apex. There are development of real-time algorithms to make physicians aware of that issue for everyday echocardiographic measurement [26]. However, foreshortening did not lead to the differences we found as all physicians used the same images. Still, the exact localization of the apex regions might be able to reduce the large scattering that was seen. Consequently, one could argue that prior to complex clinical decision e.g. implantation of a cardiac device, there should be a three-dimensional evaluation of the LVEF using automated algorithms to minimize the human factor.

Last but not least, there is much interest in the use of automated or semi-automated algorithms [4, 8, 30]. There are two obvious advantages of such modern algorithms: (1) fast image analysis and (2) less variability compared to manual contouring [4, 8, 30]. Both are relevant for clinical practice as reliable and fast image interpretation is needed. Currently, algorithms for LVEF as well as deformation imaging are available. Probably, many more will follow providing quantification of standard echocardiographic measurements and more complex analysis. Still, one of the questions will be how those software tools will be embedded in the clinical practice i.e. as fully automated solution or just providing suggestions to the cardiologists. The latter could also be seen as an educational tool leading to less observer variability.

## Conclusions

In a large group of physicians, we saw a large variability between LV contours in apical 2CH and 4CH views with larger LV volumes leading to higher LVEFs. Apparently, there were global (2CH vs. 4CH) and regional aspects (e.g. inferior vs. apical wall) as well as temporal factors (diastolic vs. systolic contours) that contributed to those differences. Also, mitral valve ring localization and apical positioning varied extensively. Overall, there were several aspects contributing to the large spreading.

## Abbreviations

ED: End-diastolic
ES: End-systolic
EF: Ejection fraction
LV: Left ventricular
2CH: Two chamber view
2D: Two dimensional
4CH: Four chamber view
